# Growth-stimulating and antioxidant effects of vanillic acid on healthy broiler chickens

**DOI:** 10.14202/vetworld.2023.518-525

**Published:** 2023-03-21

**Authors:** Galimzhan Duskaev, Marina Kurilkina, Oleg Zavyalov

**Affiliations:** Federal Research Centre of Biological Systems and Agrotechnologies of the Russian Academy of Sciences, Orenburg, Russia

**Keywords:** antioxidant status, Arbor Acres cross, blood, broiler chickens, feeding, gamma lactone, productivity, vanillic acid

## Abstract

**Background and Aim:**

Phytobiotics are a safe alternative to feed antibiotics in industrial poultry farming that increases productivity by stimulating various digestive enzymes to reduce the number of pathogenic microorganisms in the intestines and improve antioxidant status and immune response. This study aimed to evaluate the effect of vanillic acid in its pure form and in combination with gamma lactone on weight growth and the physiological parameters of broiler chickens.

**Materials and Methods:**

The studies were performed on 120 Arbor Acres broiler chickens (7 days old) that were randomly divided into four groups with five replicates (cages) and six birds per replicate. The control group was fed the basal diet (BD) only. Group I was fed the BD + gamma lactone (average dose 0.07 mL/animal/day). Group II was fed the BD + vanillic acid (average dose 0.07 mL/animal/day). Group III was fed the BD + gamma lactone + vanillic acid in a 1:1 ratio (average dose 0.07 mL/body/day). Live weight of broiler chickens for all group was assessed at the end of each period (age of 7, 14, 21, 28, 35, 42 days). At the end of the experiment (on day 42), 10 broilers with an average live weight were selected for blood sampling from the axillary vein.

**Results:**

The results showed a growth-promoting effect of vanillic acid when fed with a diet free of synthetic antioxidants. Groups I and II had increased live weights throughout the entire experiment and a significant increase at the end of the experiment (8.2%–8.5%; p ≤ 0.05) compared to the control group. Toxic effects were not found in the biochemical blood serum analyses of Groups II and III. The metabolic processes in the experimental groups were intensified, especially in the enzyme associated with amino acid metabolism (gamma-glutamyl transferase) in Groups I and III (p ≤ 0.05). Vanillic acid, whether fed alone or in combination with gamma lactone, exhibited high antioxidant activities, protected cells from oxidative damage by inducing total antioxidant, catalase, and superoxide dismutase activities (p ≤ 0.05), and reduced the level of malondialdehyde (p ≤ 0.05) measured. No significant changes in the morphological blood parameters were found.

**Conclusion:**

The use of vanillic acid alone and in combination with gamma lactone increases the digestive enzyme activities in blood plasma, increases body weight, and has a positive effect on lipid metabolism and the antioxidant status of broiler chickens. These findings indicate the significant potential use of vanillic acid and gamma lactone in poultry due to their antioxidant properties.

## Introduction

The use of antibiotics in animal feed is an effective method to increase feed digestibility, stimulate growth, and improve the quality of the products obtained [1]; however, the unjustified use of antibiotics raises concerns regarding the development of resistant bacteria, which can lead to the transfer of resistant bacteria from animals to humans [2]. These concerns have increased the need for potential antibiotic alternatives [3]. In recent years, many researchers working in farm animal feed have considered the use of phytobiotics, which are biologically active substances formed in plants, as an alternative to antibiotics [4].

The secondary bioactive compounds found in phytobiotics have properties comparable to those of synthetic antibiotics [5] and can support gut health and improve growth performance in farm animals [6]. Recent studies have shown that phytobiotics can fully replace antibiotics in the poultry industry [7]. The addition of phytobiotics to the diet of broiler chickens has a positive effect on intestinal health [8], due to their ability to modulate the dynamics of the beneficial microbiota [9], which improves feed efficiency [10] and growth rates [11, 12]. Furthermore, tannins contained in plant extracts have a positive effect on meat quality and increase the level of primary antioxidant defense enzymes in broiler chickens [13]. A positive effect of plant extracts on the biochemical properties of blood serum [14–16] and the regulation of kidney and liver functions [17, 18] have also been found.

Among the known substances of plant origin, vanillic acid may be particularly interesting. Vanillic acid, the oxidized form of vanillin, is the main active compound isolated from Chinese angelica *(Angélica archangélic*a) and green tea (*Camellia sinensis*). Green tea has a wide range of therapeutic properties in humans and laboratory animals that are associated with the ability of dietary phenol to protect biofilms and inhibit lipid peroxidation in cells [19]. In addition, vanillic acid eliminates reactive oxygen species in cells and has antimicrobial, anti-inflammatory, anticancer, and protective effects on the liver [20, 21]. Research into the potential use of this drug in the poultry industry has been limited and there is almost no data on the possible effect of the combined use of vanillic acid and other plant substances on the physiological and productive indicators of poultry.

This study aimed to evaluate the effect of vanillic acid alone and in combination with gamma lactone on the physiological parameters and productive qualities of broiler chickens.

## Materials and Methods

### Ethical approval

The study was approved by the Ethics Committee of the Orenburg State University, Orenburg, Russia (№. 2022/8 dated February 10, 2022). All animal studies were performed following the ethical standards laid down in the 1964 Declaration of Helsinki and its later amendments.

### Study period and location

The study was performed during March and April 2022 at the Experimental Biological Clinic (vivarium) (Federal Research Centre of Biological Systems and Agrotechnologies of the Russian Academy of Sciences), Orenburg, Russia.

### Experimental design

The study was performed on 120 Arbor Acres broiler chickens aged 7 days. The broiler chickens were randomly divided into four groups with five replicates (cages) each and six birds per replication. The control group was fed the basal diet (BD) only. Group I was fed the BD + gamma lactone (average dose 0.07 mL/animal/day). Group II was fed the BD + vanillic acid (average dose 0.07 mL/animal/day). Group III was fed the BD + gamma lactone + vanillic acid in a 1:1 ration (average dose 0.07 mL/body/day). Feeding and watering of the birds were performed using the group method per the recommendations of VNITIP [22].

### Weight and hematological analysis

The live weights of the broiler chickens were assessed at the end of each period (age of 7, 14, 21, 28, 35, 42 days). At the end of the experiment (on day 42), ten broilers with average body weight were selected and blood samples (3 mL) were taken from the axillary vein of the live bird. Morphological blood parameters included hemoglobin, hematocrit, erythrocytes, and leukocytes and were determined using an automatic hematology analyzer (URIT-2900 Vet Plus; URIT Medial Electronic Co., China). The biochemical analysis of the blood serum included glucose, total protein, albumin, uric acid (UA), urea (UREA), bilirubin (BIL), creatinine (CREAT), total cholesterol and its fractions alkaline phosphatase; EC 3.1.3.1, alanine aminotransferase; EC 2.6.1.2, aspartate aminotransferase; EC 2.6.1.1, phosphorus, and calcium. These measurements were obtained using an automatic biochemical analyzer (CS-T240; Dirui Industrial Co., Ltd., China); and commercial biochemical kits for veterinary medicine (DiaVetTest, Russia; and Randox, Laboratories Limited, UK).

### Statistical analysis

The Mann–Whitney U-test was used to determine significant differences. The significance level was considered p ≤ 0.05. The data were processed using the Statistica 10.0 software package (Stat Soft Inc., USA). The reference intervals were assessed using a Reference Value Advisor for MS Excel (Microsoft Inc., USA).

## Results

### Feed intake and growth indicators

The inclusion of vanillic acid and gamma lactone in the diet of experimental broiler chickens contributed to an increase in feed intake over the entire experimental period in Group I by 4.9% (214.67 g) and Group II by 4.2% (182.67 g); however, the feed consumption for the gain of 1 kg of live weight in Groups I, II, and III was lowered by 0.07 kg (4.1%), 0.08 kg (4.7%), and 0.05 kg (3.0%), respectively, compared to the control (Table-1).

**Table-1:** Feed consumption by broiler chickens against the background of separate and combined use of vanillic acid (experiment in vivarium conditions), g/head.

Indicators	Group			

	Control	I	II	III
Starter feed	1128.33	1195.67	1167.67	1062.67
Growth feed	3240.00	3387.33	3383.33	3077.33
Totally per experiment	4368.33	4583.00	4551.00	4140.00
Feed consumption for growth of 1 kg of live weight	1.69	1.62	1.61	1.64

I experimental=BD+gamma lactone (dose on average 0.07 mL/animal/day); II experimental=BD + vanillic acid (dose on average 0.07 mL/animal/day); III experimental=BD + gamma lactone + vanillic acid (1:1; average dose 0.07 mL/body/day), BD=Basal diet

In addition, the live weight dynamics differed between the experimental groups during the entire experiment (Table-2). The live weights in Groups I and II were 4.0%–8.9% and 1.4%–8.2% higher, respectively, compared to the control. Group III had the lowest live weights, which were lower than the control value by 0.3%–2.7%. Significant differences in the live weights were found at the end of the experiment (42 days), with increases of 8.5% and 8.2% (p ≤ 0.05) in Groups I and II, respectively, compared to the control.

**Table-2:** Change in live weight of broiler chickens against the background of separate and combined use of vanillic acid, g/head.

Growing period (weeks)	Group			

	Control	I	II	III
Beginning	277.3 ± 7.5	277.3 ± 7.4	277.3 ± 7.1	277.3 ± 7.7
1	604.3 ± 18.3	628.3 ± 27.5	612.6 ± 17.1	602.6 ± 25.0
2	1110.0 ± 33.9	1164.6 ± 59.3	1147.6 ± 37.7	1080.0 ± 38.3
3	1644.6 ± 41.2	1791.0 ± 95.8	1749.6 ± 53.6	1619.0 ± 49.0
4	2288.3 ± 60.5	2464.0 ± 104.5	2440.0 ± 76.3	2244.0 ± 76.2
5	2868.0 ± 85.8	3111.6 ± 80.4^a^	3103.3 ± 82.6^a^	2805.3 ± 104.4

I experimental=BD + gamma lactone (dose on average 0.07 mL/animal/day); II experimental=BD + vanillic acid (dose on average 0.07 mL/animal/day); III experimental=BD + gamma lactone+vanillic acid (1:1; average dose 0.07 mL/body/day).

a The difference is significant (p ≤ 0.05) in relation to the control group. BD=Basal diet

## Blood test results

Although no differences in the morphological parameters of the blood were found in any group compared to the control (Tables-3 and 4), differences were found between the groups when the biochemical parameters of blood serum were compared. Groups I and II showed a decrease in the level of UREA (p ≤ 0.05) and an increase in triglycerides and UA (p ≤ 0.05) compared to the control. The cholesterol levels in Groups I and II were significantly lowered by 6.8%–7.6% compared to the control (p ≤ 0.05). No statistically significant differences in the concentration of total and direct BIL were found; however, there was a downward trend in Groups II and III. In Group III, a significant increase in the level of UA by 1.3 times (p ≤ 0.05) and a decrease in the level of UREA (p ≤ 0.05) were found compared to the control. Creatinine levels were not significantly reduced in any experimental group (Table-5).

**Table-3:** Morphological parameters of the blood of broiler chickens against the background of separate and combined use of vanillic acid (M ± m [M=arithmetic mean and m=arithmetic mean error], n = 30, experiment in a vivarium).

Indicator	Group			

	Control	I	II	III
Erythrocytes, 10^12^ cells/L	3.51 ± 0.26	3.85 ± 0.06	3.75 ± 0.23	3.83 ± 0.04
Hemoglobin, g/L	114.00 ± 5.29	112.00 ± 5.57	107.50 ± 11.50	106.00 ± 0.58
Hematocrit, %	19.93 ± 0.69	19.87 ± 0.84	18.75 ± 1.95	19.20 ± 0.21
Average volume of erythrocytes, fL	108.93 ± 2.80	107.60 ± 1.14	107.90 ± 2.90	105.20 ± 1.25
Average value of hemoglobin in the cell, pg	62.00 ± 0.81	60.43 ± 1.34	61.60 ± 1.50	57.90 ± 1.91
Platelets, 10^9^ cells/L	94.33 ± 8.74	103.00 ± 9.71	81.50 ± 14.50	101.67 ± 8.76
Average platelet volume, fL	18.63 ± 0.43	18.03 ± 0.19	17.70 ± 0.40	19.13 ± 0.26

I experimental=BD + gamma lactone (dose on average 0.07 mL/animal/day); II experimental=BD + vanillic acid (dose on average 0.07 mL/animal/day); III experimental=BD + gamma lactone+vanillic acid (1:1; average dose 0.07 mL/body/day). BD=Basal diet

**Table-4:** The content of white blood cells in broiler chickens against the background of separate and combined use of vanillic acid (M ± m [M=arithmetic mean and m=arithmetic mean error], n = 30, experiment in a vivarium).

Indicator	Group			

	Control	I	II	III
Leukocytes, 10^9^ cells/L	41.20 ± 4.94	41.03 ± 6.18	36.65 ± 4.25	37.03 ± 1.73
Lymphocytes, %	54.17 ± 1.59	52.90 ± 1.52	55.25 ± 0.55	55.50 ± 0.31
Monocytes, %	7.73 ± 0.10	8.50 ± 0.35^a^	7.55 ± 0.25	7.93 ± 0.02^a^
Granulocytes, %	38.10 ± 1.02	39.13 ± 1.20	37.20 ± 0.30	36.57 ± 0.32

I experimental=BD + gamma lactone (dose on average 0.07 mL/animal/day); II experimental=BD + vanillic acid (dose on average 0.07 mL/animal/day); III experimental=BD + gamma lactone+vanillic acid (1:1; average dose 0.07 mL/body/day).

a The difference is significant (p ≤ 0.05) in relation to the control group. BD=Basal diet

**Table-5:** Biochemical parameters of blood of broiler chickens against the background of separate and combined use of vanillic acid (M ± m [M=arithmetic mean and m=arithmetic mean error], n = 30, experiment in a vivarium).

Indicator	Group			

	Control	I	II	III
GLU, mmol/L	13.70 ± 0.67	15.04 ± 0.39	14.95 ± 0.19	15.12 ± 0.26
TP, g/L	44.19 ± 1.97	42.38 ± 0.82	40.18 ± 2.70	43.40 ± 2.37
ALB, g/L	20.00 ± 1.15	20.67 ± 0.88	18.67 ± 2.19	20.00 ± 1.53
BIL total, µmol/L	0.58 ± 0.12	0.76 ± 0.21	0.49 ± 0.07	0.35 ± 0.08
BIL direct, µmol/L	0.33 ± 0.05	0.33 ± 0.06	0.30 ± 0.03	0.26 ± 0.02
Cholesterol, µmol/L	3.68 ± 0.11	3.43 ± 0.05	3.40 ± 0.08^a^	3.69 ± 0.20
Triglycerides, µmol/L	0.22 ± 0.01	0.35 ± 0.05a	0.25 ± 0.01^a^	0.22 ± 0.01
UREA, mmol/L	0.57 ± 0.06	0.37 ± 0.05a	0.43 ± 0.03^a^	0.43 ± 0.12^a^
CREAT, µmol/L	20.97 ± 0.23	20.03 ± 1.63^a^	19.33 ± 1.20	19.57 ± 1.47
UA, µmol/L	87.37 ± 3.87	110.03 ± 8.55^a^	96.00 ± 1.73^a^	117.77 ± 12.08^a^
Ca, µmol/L	3.67 ± 0.08	3.02 ± 0.33	3.65 ± 0.28	3.63 ± 0.09
P, mmol/L	10.86 ± 0.37	10.01 ± 0.39	7.28 ± 1.80	10.03 ± 0.26
Iron, µmol/L	15.67 ± 0.99	26.47 ± 5.42	29.93 ± 7.19	24.30 ± 4.64

I experimental=BD + gamma lactone (dose on average 0.07 ml/animal/day); II experimental=BD + vanillic acid (dose on average 0.07 mL/animal/day); III experimental=BD + gamma lactone+vanillic acid (1:1; average dose 0.07 ml/body/day).

a The difference is significant (p ≤ 0.05) in relation to the control group. BD=Basal diet, GLU=Glucose, TP=Total protein, ALB=Albumin, BIL=Bilirubin, UREA=Urea, CREAT=Creatinine, UA=Uric acid, Ca=Calcium, P*=*Phosphorus

An increase in the enzyme associated with amino acid metabolism (gamma-glutamyl transferase) was found and was especially obvious in Groups I and III (p ≤ 0.05). Group I had an increase in amylolytic enzyme a-amylase activities (p ≤ 0.05) compared to the control. A high concentration of the lipase enzyme was also established in Groups I and II (p ≤ 0.05) compared to the control. Superoxide dismutase and catalase were increased in Groups I and III compared to the control by 2.6%–10.6% (p ≤ 0.05) and 50%–66.7%, respectively (p ≤ 0.05). All experimental groups had a significant decrease in the concentration of malondialdehyde in the blood, as an indicator of lipid peroxidation in birds (p ≤ 0.05) (Table-6).

**Table-6:** The activity of blood serum enzymes and indicators of the antioxidant status of broiler chickens against the background of separate and combined use of vanillic acid in the diet (M ± m [M=arithmetic mean and m=arithmetic mean error], n = 30, experiment in a vivarium).

Indicator	Group			

	Control	I	II	III
alanine aminotransferase, U/L	16.53 ± 4.27	11.50 ± 0.81	11.47 ± 4.22	23.23 ± 1.33
Aspartate aminotransferase, U/L	440.33 ± 57.02	349.47 ± 48.47	483.57 ± 32.94	472.87 ± 7.44
Gamma-glutamyl-transferase, U/L	25.00 ± 0.70	26.67 ± 0.46^a^	22.67 ± 1.73	26.67 ± 0.45^a^
ALP, U/L	1904.3 ± 35.24	1879.67 ± 153.96	1958.67 ± 243.53	1993.0 ± 191.37
a-amylase, U/L	83.33 ± 40.48	271.00 ± 81.24^a^	72.00 ± 38.42	84.00 ± 36.12
p-amylase, U/L	254.30 ± 118.65	461.77 ± 120.25	160.67 ± 79.73	442.70 ± 84.99
Lipase, U/L	4.83 ± 2.20	15.30 ± 3.34^a^	14.17 ± 2.87^a^	6.40 ± 1.46
Superoxide dismutase, %	68.07 ± 0.74	70.18 ± 0.52^a^	69.82 ± 0.24^a^	75.26 ± 2.69^a^
Malondialdehyde, µM/L	313.91 ± 20.21	154.95 ± 65.73^a^	240.65 ± 28.12^a^	253.08 ± 19.20^a^
Catalase, µM H_2_O_2_/Lhmin	0.06 ± 0.01	0.10 ± 0.02^a^	0.07 ± 0.02	0.09 ± 0.01^a^

I experimental=BD + gamma lactone (dose on average 0.07 mL/animal/day); II experimental=BD + vanillic acid (dose on average 0.07 mL/animal/day); III experimental=BD + gamma lactone + vanillic acid (1:1; average dose 0.07 mL/body/day).

a The difference is significant (p ≤ 0.05) in relation to the control group. BD=Basal diet, ALP=Alkaline phosphatase

The introduction of gamma lactone and vanillic acid in combination with gamma lactone in the diet of broiler chickens contributed to the stimulation of non-specific resistance due to an increase in the proportion of monocytes in the leukogram. The maximum monocyte content was found in Group I (p ≤ 0.05), which may indicate an increase in the functional activities of the cellular factors of immunity.

## Discussion

The growth-stimulating effect of vanillic acid and gamma lactone was revealed in our study, is likely due to the beneficial effect of these compounds on the digestive system of birds, which includes a restoration of the microbiota balance and improvements in the absorption of nutrients [23]. Our results are consistent with the findings of Salaheen *et al*. [24], who found that the phenolic extracts of blueberry (*Vaccinium corymbosum*) and blackberry (*Rubus fruticosus*) plants can modulate the gut microbiome and increase weight gain [24]. A similar effect was found with the addition of cumin, basil, lemon, laurel, sage, oregano, thyme, and tea essential oils [25], as well as the addition of carvacrol, cinnamic aldehyde, and capsicum resins [26, 27] and the herbal mixture of teknaroma [28]. In addition to the stimulation of beneficial intestinal microflora in broiler chickens, some experiments found a decrease in the level of pathogenic microorganisms. This has been particularly shown for cinnamaldehyde essential oils [29].

The positive effects of vanillic acid and gamma lactone on the growth of broiler chickens found in this experiment might be associated with the previously discovered antimicrobial properties of phenolic compounds and the similar properties of gamma lactone [30] based on their ability to block quorum sensing (QS) signaling systems and inhibit the formation of biofilms in clinically significant pathogens. In particular, plant extracts containing phenolic compounds, quinones, flavonoids, alkaloids, terpenoids, and polyacetylenes are presented as effective and safe substances that inhibit QS systems [31]. For example, Poli *et al*. [32] identified a clear relationship between *Chromobacterium violaceum* and anti-QS activities. A 50 mg/mL concentration of coumarin inhibits the formation of an *E. coli* biofilm by more than 80% without affecting bacterial growth [33], which activates the beneficial microflora, contributes to a more complete breakdown and absorption of substances, and increases the productivity of the bird [34].

Many phenolic compounds, including vanillic acid, serve as effective antimicrobial agents [35, 36]. Although the mechanisms of these antimicrobial properties are not fully understood, it is hypothesized that they are caused by the destabilization of the microbial cell surface and cytoplasmic membranes [37–39], which can lead to irreversible damage to the cell wall and various intracellular organelles, the coagulation of cellular organs, and the inhibition of intracellular enzymes. In particular, hydrophobic phenolic compounds bind to the lipid bilayer of the microbial cell membrane, causing structural damage and a loss of integrity [40], which leads to the formation of pores, the leakage of intracellular components into the extracellular space, and the functional dysregulation of proteins [41, 42]. In addition, the phenol ring can act as a transmembrane cation carrier due to its hydroxyl group and irreplaceable double bonds [43]. Furthermore, phenolic compounds can interact with intracellular components and DNA after the destruction of the cell wall and penetration into the cell [44, 45]. Destruction of intracellular membranes causes the release of free radicals, which can lead to DNA damage and lipid oxidation. As an adaptive response to this phenolic attack, microbes modify their gene expression to reduce aerobic metabolism and increase the production of antioxidants and DNA-repair enzymes [46–48]. The suppression of aerobic metabolism alone limits microbial motility and biofilm formation, which are conditions conducive to survival. Recent study has shown that the use of phenolic compounds for 6 weeks results in an increase in broiler chicken weights by 9.5% and a higher ratio of firmicutes to bacteroidetes species in the microbiome of broiler chickens [24].

In our experiment, the synergistic effect of vanillic acid and gamma lactone (Group III) on growth was less pronounced than the individual addition of vanillic acid (Group II) and gamma lactone (Group I) to the diet of broiler chickens. The results of our studies showed that the separate use of vanillic acid and gamma lactone in the diet of broilers led to a significant decrease in cholesterol levels and an increase in triglycerides and UA. These data are consistent with previous experiments on Arbor Acres broilers that demonstrated increases in the blood levels of UA and triglycerides when coumarin was included in the diet [49]. Another study demonstrated a decrease in serum cholesterol levels in groups treated with antibiotics and essential oils [50]. Similar effects were also obtained when cinnamon essential oils were added to the diet [51].

Other studies indicated an opposite effect on cholesterol synthesis after the inclusion of phytobiotics due to the bioactive compounds present in essential oils, such as phenol, cineole, borneol, citral, menthone, geraniol, menthol, fenchyl alcohol, fenchone, and ionone, which are required to inhibit the activity of 3-hydroxy-3-methylglutarylcoenzyme A reductase in the liver, the most important enzyme for cholesterol synthesis [52]. In our experiment, vanillic acid, both alone and in combination with gamma lactone, decreased the content of the bile pigment, but the changes were not significant. A decrease in the level of direct BIL in the blood serum in this study indicates normal BIL metabolism and the lack of a toxic effect of vanillic acid, which is consistent with the previous studies [53, 54]. The level of CREAT and UREA can be used to judge the functional state of the kidneys. In our experiment, the introduction of vanillic acid and gamma lactone significantly decreased UREA and simultaneously decreased CREAT. It is likely that this effect is associated with the intensification of biochemical pathways and the transition of this metabolite to creatine phosphate.

Vanillic acid increases the activation of glutathione peroxidase, superoxide dismutase, and catalase and scavenges free radicals [55]. The antioxidant activities of vanillic acid are primarily due to its phenolic properties, since phenols can act as electron donors or acceptors depending on the redox state of the system [55]. Phenolic components are structural fragments responsible for the antioxidant activity of many other natural bioactive substances [56]. The phenolic compounds in vanillic acid are considered effective antioxidants due to their ability to deactivate and stabilize free radicals by incorporating them into the aromatic ring and absorbing ultraviolet light [57, 58]. Unlike synthetic phenolic antioxidants [59, 60], natural phenolic compounds have a beneficial effect on health by protecting against chronic diseases associated with oxidative stress [61, 62]. The increase in the level of anti-acid protection and decrease in the degree of lipid peroxidation in the broiler chickens of Groups I, II, and III in this study were indicated by the positive dynamics of the catalase and superoxide dismutase enzyme activities, which was found together with a significant decrease in the level of malondialdehyde. Similar results have been obtained using gamma-octalactone isolated from *Eucalyptus viminalis* leaf extract and other plant extracts [30, 63–65].

## Conclusion

The positive effect of vanillic acid alone and in combination with gamma lactone on antioxidant properties obtained in this study may support our hypothesis that the improvement in growth performance is associated with better chick health, although the improvement in growth performance of the administered compounds was not as significant as it was for the blood parameters. The present study showed how the administration of vanillic acid alone and in combination with gamma lactone to broiler chicken feed can improve immunity and antioxidant properties. Further research in this direction should involve studying the effects of vanillic acid in combination with other substances of plant origin on the intestinal microbiome in poultry.

## Authors’ Contributions

GD: Supervised the study and developed experimental methods. MK: Mathematical processing of experimental data and drafted the manuscript. OZ: Conducted the experiments and analyzed and interpreted data. All authors have read, reviewed, and approved the final version of the manuscript.
